# New Possibilities on the Horizon: Genome Editing Makes the Whole Genome Accessible for Changes

**DOI:** 10.3389/fpls.2019.00525

**Published:** 2019-04-24

**Authors:** Katharina Kawall

**Affiliations:** Fachstelle Gentechnik und Umwelt, Munich, Germany

**Keywords:** genome editing, CRISPR/Cas, base editing, mutations, mutagenesis, genetic variation, meiotic recombination, DNA mismatch repair

## Abstract

The emergence of new genome editing techniques, such as the site-directed nucleases, clustered regulatory interspaced short palindromic repeats (CRISPRs)/Cas9, transcription activator-like effector nucleases (TALENs), or zinc finger nucleases (ZFNs), has greatly increased the feasibility of introducing any desired changes into the genome of a target organism. The ability to target a Cas nuclease to DNA sequences with a single-guide RNA (sgRNA) has provided a dynamic tool for genome editing and is naturally derived from an adaptive immune system in bacteria and archaea. CRISPR/Cas systems are being rapidly improved and refined, thereby opening up even more possibilities. Classical plant breeding is based on genetic variations that occur naturally and is used to select plants with improved traits. Induced mutagenesis is used to enhance mutational frequency and accelerate this process. Plants have evolved cellular processes, including certain repair mechanisms that ensure DNA integrity and the maintenance of distinct DNA loci. The focus of this review is on the characterization of new potentials in plant breeding through the use of CRISPR/Cas systems that eliminate natural limitations in order to induce thus far unachievable genomic changes.

## Introduction

Genetic variation is a prerequisite of both natural and artificial selection. It allows populations to adapt to changing environmental conditions from generation to generation. Genetic variation occurs naturally through spontaneous mutations, processes during meiosis, and gamete combinations during fertilization or is induced by mutagenesis. These processes generate natural variation and are undirected but cannot be considered to be purely random.

A mutation is any change in genetic material that does not originate from the crossing of two individuals. Mutations can occur spontaneously or can be induced by external factors. DNA damage, which subsequently leads to the manifestation of mutations, is caused, for example, internally by mistakes during DNA replication, or can be induced by environmental factors, such as irradiation (e.g., UV light) or mutagenic substances. The occurrence of new mutations is not purely random since certain repair mechanisms, the local composition of the DNA sequence, and the chromatin state influence the retention of preceding DNA damage. DNA replication is a highly accurate biological process, but mistakes can occasionally occur when the DNA polymerase inserts a wrong base in the newly synthesized (daughter) strand. Most DNA polymerases directly correct mismatches through their proofreading function during DNA polymerization by sensing base mispairing. Nevertheless, some mismatches escape proofreading and are repaired after replication *via* DNA mismatch repair (MMR; Kunkel and Erie, 2015).

Meiosis is a fundamental biological process resulting in the reassortment and recombination of the genetic content received from the parents. In sexually reproducing organisms, meiosis generates haploid germ cells, which can fuse with a germ cell from another individual to generate a diploid zygote that develops into a new individual. In plants, the diploid state is referred to as the sporophyte generation, and the haploid stage as the gametophyte generation. Both recombination during meiosis and chromosome reassortment of parental DNA guarantee that each gamete generated will receive a unique combination of alleles. Significantly, at least one recombination event occurs between a maternal and a parental chromatid leading to an exchange of genetic material, thereby generating genetic variation. The genomic regions of the recombination events are not purely random but, rather, occur at predetermined sites correlating with specific genomic features such as epigenetic marks and DNA sequence motifs. Genes that are genetically linked in low recombinogenic genomic regions are inherited collectively; genes located at high genetic distance or between recombinational hot spots are more likely to be separated by crossing over.

Genome editing is a site-directed process that enables the editing of DNA sequences by using technologies such as clustered regulatory interspaced palindromic repeats/CRISPR associated (CRISPR/Cas), transcription activator-like effector nuclease (TALEN), or zinc finger nuclease (ZFN). The focus of this review is on CRISPR/Cas since it is one of the most powerful and promising genome editing techniques shaping the future of biotechnology.

Among other applications, CRISPR/Cas is used to edit DNA and RNA or to alter the epigenetic landscape of chromatin. The simplicity and flexibility of the system come from its targeting component, the guide RNA (gRNA), which directs the endonuclease Cas9 to the intended DNA region. Given that a reference genome exists, the gRNA can easily be designed and produced (Doudna and Charpentier, 2014). This elegant molecular toolset can be used in plant breeding to develop varieties with new genetic combinations that were not possible to establish thus far. Genome editing is expanding the options for editing the genome beyond what is possible naturally; it is also opening up inaccessible areas of the genome. Changes made through genome editing are not in any case identical to spontaneously occurring mutations or to products from genetic recombinations. As genome editing is a targeted biotechnology, it is misleading to conclude that it enhances genetic variation. The aim of this review is to give an overview of the promising new possibilities of genome editing (focusing on CRISPR/Cas9), especially in plant breeding; and how genome editing bypasses natural limitations to introduce changes in any chosen part of the genome.

BOX 1 **Technical characterization of CRISPR/Cas.**

CRISPR/Cas was discovered as an adaptive immune system in bacteria (Mojica et al., 2005; Barrangou et al., 2007) and successively adapted as a molecular biotechnology tool. CRISPR/Cas allows the precise targeting of an endonuclease (e.g., Cas9) to specific genomic regions *via* a gRNA (Jinek et al., 2012; Doudna and Charpentier, 2014). The gRNA is designed individually depending on the genomic loci that are to be altered. Cas9 interacts with the gRNA and binds and slides along the DNA to screen for the corresponding target sequence. A nuclease-specific protospacer adjacent motif sequence (PAM sequence) adjacent to the target sequence is the initial trigger that stops the nuclease from sliding along the DNA (Jinek et al., 2013; Sternberg et al., 2014). If the gRNA complementarily binds to the target sequence by Watson-Crick base pairing, the Cas9-gRNA complex undergoes a conformational change, which generates an activated nuclease capable of tightly binding and introducing a DNA double-strand break (DSB) at the target sequence (Nishimasu et al., 2014). The DSBs subsequently activate cellular repair mechanisms in the cell that mainly include non-homologous end joining (NHEJ) and homology-directed repair (HDR; Rudin et al., 1989; Plessis et al., 1992; Rouet et al., 1994a,b; Choulika et al., 1995). The NHEJ pathway is error prone and usually introduces base insertions or deletions (indels) at the DNA break sites (Gorbunova and Levy, 1999). These indels can generate frameshift mutations or disrupt important functional domains, which, for example, disturb the functions of the target genes (Mali et al., 2013). The HDR pathway is a DNA repair mechanism that can be utilized to introduce nucleotide substitutions and insertions of desired sequences at the target sites or to replace DNA sequences by using exogenous DNA donor templates (Shan et al., 2013; Svitashev et al., 2015; Zhao et al., 2016). Thus, the CRISPR/Cas9 system provides a powerful tool for sequence-specific genome editing, including gene knockouts, gene knockins, and site-specific sequence alterations (Doudna and Charpentier, 2014; Hsu et al., 2014; Sander and Joung, 2014). A more detailed description of the CRISPR/Cas system is discussed in depth elsewhere (Wang et al., 2016; Jiang and Doudna, 2017; Langner et al., 2018).

## CRISPR/Cas Allows the Simultaneous Modification of Multiple Genomic Regions

CRISPR/Cas is a powerful toolset to genetically engineer the genome of target organisms. A detailed description of CRISPR/Cas is summarized in Box 1. One major advantage of CRISPR/Cas9 compared to conventional plant breeding is its ability to combine multiple gRNAs to introduce changes at different target sites of the genome simultaneously or successively (Mao et al., 2013; Ma et al., 2015). This CRISPR/Cas application is called multiplexing and has already been used to change many major crop plants such as wheat (Wang et al., 2018), tomato (Li et al., 2018a,b), maize (Qi et al., 2016), soya (Cai et al., 2018), and rice (Shen et al., 2017). Many plant traits, e.g., the response to abiotic stress, are dependent on the expression of multiple genes, which need to be edited in a well-orchestrated manner. New CRISPR/Cas9 systems have been developed for multiplexing approaches in plants allowing a systematic study of gene families or metabolic pathways (Xing et al., 2014; Xie et al., 2015). For example, Li et al. previously generated tomato plants with increased lycopene content using a multiplexing approach. They used CRISPR/Cas9 to manipulate multiple genes associated with the carotenoid metabolic pathway of tomatoes in order to increase the lycopene content in fruits and also to explore the roles of these key genes in carotenoid metabolism (Li et al., 2018b). Zhang et al. previously generated a multiplex CRISPR/Cas9 platform to co-express six gRNA modules in one vector in *Arabidopsis thaliana* (Zhang et al., 2016). They targeted six of 14 PYR/PYL gene family members of ABA receptor genes in a single transformation event. The mutagenesis frequency for the six individual PYL targets ranged from 13 to 93% in T1 lines, and one transgenic line was identified that contained mutations in all six PYL genes (Zhang et al., 2016). Further outstanding work that demonstrates the potential of CRISPR/Cas9 is a recently published paper by Sanchez-Leon et al., who used two gRNAs in wheat to target CRISPR/Cas9 to a conserved region in α-gliadin genes (Sanchez-Leon et al., 2018). α-gliadin is one component of gluten proteins in wheat that triggers an autoimmune disorder called coeliac disease in genetically predisposed individuals through ingestion. Up to 35 copies of the 45 different α-gliadin genes were simultaneously mutated in one of the generated lines leading to a reduction in immunoreactivity by 85% (Sanchez-Leon et al., 2018). So far, efforts in conventional plant breeding and mutagenesis have failed to obtain low-gluten wheat varieties due to the high copy number of the α-gliadin genes. RNAi has already been used to mediate the downregulation of gliadin genes efficiently (Gil-Humanes et al., 2010). But, in contrast to the RNAi gliadin knockout lines, CRISPR/Cas knockouts are stable and heritable mutations that do not contain any transgenes in their genome.

## Further Development of Cas Nucleases Broadens the Spectrum of Genomic Alterations

Targeting Cas9 to a DNA site is easy to achieve by generating a new gRNA that is complementarily binding to the desired DNA target site adjacent to a corresponding PAM sequence. Initially, the CRISPR/Cas9 system could not be used to target any sequence in the genome, as it requires a nuclease-specific PAM sequence. The PAM sequence of Cas9 derived from *Streptococcus pyogenes* (SpCas9) is the 5′-NGG-3′ motif (Doudna and Charpentier, 2014; Hsu et al., 2014; Sander and Joung, 2014; Lee et al., 2016). Although the PAM sequence is frequently present in the genome, the requirement of a PAM sequence for the activity of Cas9 limits the numbers of potential genomic target sequences, especially in AT-rich regions. Cas nucleases from different bacteria have varying PAMs and properties, which further expands the range of targetable genomic sequences (Esvelt et al., 2013; Ran et al., 2015). Cas9 orthologs, for example, from *Staphylococcus aureus* (SaCas9), *Staphylococcus thermophilus* (StCas9), and *Neisseria meningitidis* (NmCas9) have different PAM sequences to the original *S. pyogenes* SpCas9. SaCas9 requires a 5′-NNNNGATT-3′, StCas9 a 5′-NNGGAA-3′, and NmCas9 either 5′-NNNNGATT-3′ or 5′-NNNNGCTT-3′ PAM sequence (Esvelt et al., 2013). The endonuclease Cpf1 is a CRISPR endonuclease discovered in *Prevotella* and *Francisella* bacteria, which offers an alternative CRISPR system for genome editing and has already been used in various organisms, including plants (Zetsche et al., 2015; Zhang et al., 2016; Kim et al., 2017; Tang et al., 2017; Xu et al., 2017). Cpf1 has several distinct features compared to Cas9: first, the nuclease utilizes a thymidine-rich PAM site 5′-TTTN-3′ enabling new targeting possibilities (Yamano et al., 2016). Recent work in human cells has revealed that Cpf1 can be modified to recognize alternative PAM sequences (Gao et al., 2017). Second, in contrast to Cas9 generating blunt-ended DSBs, Cpf1 creates 4–5 nucleotide long sticky ends enhancing the efficiency of DNA insertions and the specificity during NHEJ or HDR repair (Zetsche et al., 2015).

SpCas9 has also been engineered to generate new versions of Cas9 in order to recognize various PAM sequences improving the targeting capacity of the nuclease (Kleinstiver et al., 2015a,b; Hu et al., 2018; Nishimasu et al., 2018). Recently, mutants in the PAM recognition domain of SpCas9 and other adjacent residues were generated resulting in the engineering of a SpCas9-TG variant that is able to recognize PAM sequences with increased versatility across genomes (Nishimasu et al., 2018). Nevertheless, the activity of SpCas9-TG needs to be further improved as in comparison to wild-type SpCas9, the cleavage activity of SpCas9-TG at NGG sites is lower. In another approach that relies on the phage-assisted continuous evolution of SpCas9, an evolved PAM SpCas9 variant (xCas9) was generated that recognizes a wide range of different PAM sequences including NG, GAA, and GAT (Hu et al., 2018).

A catalytically inactive “dead” Cas9 variant (dCas9) was developed and fused to diverse functional domains for various applications such as base editing (Komor et al., 2016), editing of epigenetic modifications (McDonald et al., 2016; Huang et al., 2017), or transcriptional silencing (Larson et al., 2013; Qi et al., 2013). Base editing is a form of genome editing that enables the direct, irreversible conversion of a specific DNA base into another at a targeted genomic locus without requiring DSBs, homology-directed repair (HDR), or donor DNA templates (Gaudelli et al., 2017; Hess et al., 2017). Base editors induce lower levels of unwanted insertions and deletions when compared to standard genome editing (Komor et al., 2016; Rees et al., 2017), but they have the ability to change all the target nucleotides within an editing window of five base pairs (Komor et al., 2016). New base editing systems are being established in order to remove this limitation and to increase the specificity (Gehrke et al., 2018). Another Cas-variant from *Leptotrichia wadei* (LwaCas13a) was identified (Abudayyeh et al., 2016) and adopted for precise editing of RNA (Abudayyeh et al., 2017). Cas13 enzymes can mediate precise RNA cleavage, which was already used for targeted knockdown of endogenous transcripts in rice protoplasts with comparable levels of knockdown as RNA interference and improved specificity (Abudayyeh et al., 2017). A catalytically inactive Cas13 enzyme (dCas13) can be used for RNA editing to direct adenosine deaminase acting on RNA 2 (ADAR2) to mRNA (Cox et al., 2017).

These new adaptations of the classical CRISPR/Cas9 system show how powerful this technology is and how further improvements might lead to a growing number of possibilities.

## Genetic Variation Through Meiotic Recombinations

Meiosis is a fundamental biological process that occurs in sexually reproducing organisms generating genetic variation by recombining the genetic content received from the parents and is a highly orchestrated molecular process. Meiosis-specific expression of the endonuclease SPO11 (meiotic recombination protein SPO11) causes many DNA double-strand breaks (DSBs) along the paired chromosomes during Prophase I (Keeney et al., 1997; Keeney, 2008). A subset of these break sites are repaired through recombination pathways that lead to a reciprocal exchange of DNA between homologous chromosomes called crossing overs (COs) generating new allele combinations and increasing genetic variation (Szostak et al., 1983). The majority of meiotic DSBs are repaired without an exchange of flanking regions, which are called non-crossing overs (NCOs; Wang and Copenhaver, 2018).

In *Arabidopsis thaliana*, approximately 150–250 DSBs occur during meiosis (Mercier et al., 2015; Lambing et al., 2017). The repair of these DSBs results in the formation of approximately 10 COs in one generation (Yang et al., 2012). There is strong evidence for the existence of recombinational CO hot and cold spots, with some regions entirely devoid of COs and others containing large numbers of COs concentrated within a few kilobases, suggesting an uneven distribution of COs in the genome of plants (Giraut et al., 2011; Lu et al., 2012; Salome et al., 2012; Drouaud et al., 2013; Si et al., 2015). COs in plants are enriched in euchromatin at the chromosome scale and in proximity to gene promoters and terminators at the fine scale (Horton et al., 2012; Choi et al., 2013; Drouaud et al., 2013; Wijnker et al., 2013; Shilo et al., 2015). In plants, COs correlate with the histone mark H3K4me3 and the histone variant H2A.Z, which are associated at promoters with an A-rich DNA motif, while the CNN repeat and the CTT repeat motifs are preferentially associated with genes and can be suppressed by the acquisition of heterochromatic modifications, such as DNA methylation and H3K9me3 (Choi et al., 2013; Drouaud et al., 2013; Wijnker et al., 2013; Shilo et al., 2015; Yelina et al., 2015).

The induction of DSBs by SPO-11 is strongly influenced by AT-sequence richness and low-nucleosome density regions, most likely allowing SPO-11 increased access to the DNA (Choi et al., 2018). DNA regions with highly repetitive sequences undergo little or no meiotic recombination. Repeat-rich regions are located in centromeres, telomeres, transposable elements, ribosomal RNA, and transfer RNA loci (Chen et al., 2008; Termolino et al., 2016). Also, in the large genomes of many grass species, including wheat, the majority of the chromosomes consist of non-recombining expanses of heterochromatin, which can cause significant limitations for breeding (Lambing et al., 2017).

In cases where certain plant genes have a low genetic distance, meaning they are located in close proximity in low or non-recombinogenic regions, the probability of separating them is highly unlikely during breeding. Genetic distance is measured in centimorgan defining the distance between genomic loci on a chromosome. A centimorgan is defined as the distance between chromosome positions for which the expected average number of intervening chromosomal crossovers in a single generation is 0.01. If a gene of breeding interest is linked to a gene with adverse effects on, e.g., yield or fruit shape, those genes might not be separable through conventional breeding. Breeders often have to work with blocks of linked genes during backcrossing as they cannot directly replace single alleles with another in most species (Young and Tanksley, 1989). Previous genomic analyses in tomato plants have indicated that the linkage drag associated with genome segmentation covers nearly 25.6% of the assembled genome, limiting further improvement *via* genetic recombination at meiosis during breeding (Lin et al., 2014). Also in barley, a high proportion of genes fall into linkage blocks that are refractory to recombination as most genes are located within the pericentromeric region and recombination predominantly occurs in distal regions of the chromosomes (International Barley Genome Sequencing et al., 2012; Phillips et al., 2013, 2015; Baker et al., 2014). Separation of linked genes is challenging and labor intensive using classical breeding methods. Genome editing enables access and direct allele replacement for all genes in a fast and easy way. If a target gene is linked to a gene that encodes an undesirable trait, genome editing can be used to remove the undesirable gene without having to do extensive backcrossing. However, target genes associated with undesirable traits that cause linkage drag need to be identified in order to perform genome editing. Recently, CRISPR/Cas was used to overcome a linkage drag effect in tomato plants by editing the jointless2 (J2) gene (Roldan et al., 2017; Soyk et al., 2017). J2 is essential for the formation of the abscission zone that is present in the pedicel of tomato plants (Mao et al., 2000). J2 mutants fail to develop the pedicel abscission zone, which is the zone where ripe fruits break off and fall off the plant. J2 mutant fruits fall off the plants without the pedicel remaining attached to the fruit, which prevents damage to other fruits by the stem piece (Ito and Nakano, 2015). The J2 mutation is also associated with misshaped fruits, which is a less desirable trait. These misshaped fruits might be the result from another gene closely located to the J2 gene. Using CRISPR/Cas9, J2 mutants were generated without associated linkage drag effects on fruit morphology (Roldan et al., 2017).

## Cellular Repair Mechanisms Shape the Mutational Spectrum and the Frequency of Spontaneous Mutations

Evolutionary pressure enriches mutations at certain genomic regions over many generations (Ossowski et al., 2010). Besides this selection process that is highly dependent on environmental factors, a bias exists toward the location of *de novo* mutations in the genome. The induction of DNA damage is the initial trigger for the formation of new mutations. This can occur externally through environmental factors such as UV sunlight, or exposure to mutagenic substances, or internally through mistakes during replication, or by metabolic byproducts (e.g., reactive oxygen species; Tripathy and Oelmuller, 2012; Manova and Gruszka, 2015). DNA damage caused by these external and internal factors includes base lesions, intra- and interstrand cross-links, and DNA single- and double-strand breaks (Lindahl, 1993). Many spontaneous mutations arise from errors of the DNA polymerase during DNA replication primarily resulting in base mispairing (Kunz et al., 1998; Stuart et al., 2000). Most DNA polymerases correct mismatches by their proofreading function directly during replication. Nevertheless, some mismatches escape proofreading and are repaired after the replication by the DNA mismatch repair (MMR; Kunkel and Erie, 2015). The MMR system is important for genomic integrity and therefore highly conserved in all living organisms from bacteria to higher eukaryotes. In eukaryotes, a set of MutS homolog (MSH) genes and MutL homolog (MLH) genes assemble the MMR system harboring specialized functions during replicative repair (Culligan et al., 2000). Further, in eukaryotes, the exact recognition mechanisms are not yet fully understood, but it is evident that they involve a tight interplay between replication and MMR (Jiricny, 2013). The corresponding proteins are incorporated in different heterodimeric complexes like MSH2/MSH6 or MSH2/MSH3, which recognize certain DNA structures or DNA damage. Generally, the MSH heterodimers recognize DNA mismatches and bind the DNA, whereas the actual repair mechanism is subsequently initiated by recruitment of the MLH heterodimer (Culligan and Hays, 2000). MMR has been implicated in the removal of incorrectly paired nucleotides and UV-induced photolesions from the genome of higher plants (Culligan and Hays, 2000; Lario et al., 2011).

In *Arabidopsis thaliana*, MMR deficiency was sufficient to significantly increase the mutation rate without any mutagenic treatment (Chao et al., 2005). The inactivation of MMR was additionally shown to enhance mutagenesis with reduced associated toxicity, as lower doses of mutagens were sufficient to obtain desired traits in an MMR deficient background (Hoffman et al., 2004). Genomes of crop species such as rice (Xu et al., 2012), tobacco (Van Marcke, 2013), and tomato (Tam et al., 2011) have already been altered by a manipulated MMR system.

Recently, the mutational spectrum of MMR-deficient *Arabidopsis thaliana* mutation accumulation (MA) lines lacking the core subunit MSH2 of the MMR complex was investigated (Belfield et al., 2018). MA lines exhibit an increased frequency of spontaneous mutations and a different molecular mutational spectrum compared to MMR-proficient strains (Hoffman et al., 2004; Belfield et al., 2018). In this approach, only strongly deleterious mutations are lost, whereas others (weakly deleterious, neutral, and advantageous) accumulate over time (Belfield et al., 2018). Surprisingly, the authors found in genome-wide analyses that in *Atmsh2–1* mutant plants, the relative frequency of single-nucleotide variations (SNVs) in genic regions (in CDS introns and UTRs) is significantly increased (relative to wildtype), whereas that of non-genic regions (intergenic, TE) is reduced. Thus, mutations are spread randomly throughout the genome of MMR-deficient strains, while they are non-randomly distributed throughout the genome of MMR-proficient strains. This is a clear indication that MMR preferentially protects genes, rather than non-genic regions of the genome, from mutations. How targeting of MMR to genic regions of the genome works in plants still remains to be answered.

There is good evidence that the recruitment of MMR and the protection of genic regions are a conserved mechanism as similar results were also found in *Escherichia coli* (Lee et al., 2012; Foster et al., 2018; Niccum et al., 2018), *Saccharomyces pombe* (Sun et al., 2016), and human cell lines (Supek and Lehner, 2015; Huang et al., 2018). In human tumors, after the inactivation of MMR, mutations are no longer enriched in late replicating heterochromatin relative to early replicating euchromatin (Supek and Lehner, 2015). Active genes are located in euchromatin and replicate early during S-phase. A properly functioning MMR in early replicating euchromatin appears to be an evolutionarily conserved mechanism to protect genic regions.

It was also shown in human cells that the recruitment of MMR to genic regions during replication correlates with the histone mark H3K36me3 (Li et al., 2013). H3K36me3 is highly enriched in actively transcribed open chromatin and abundant in exonic regions (Wagner and Carpenter, 2012). Beside the function of MMR and H3K36me3 during replication, it was also recently shown in human HeLa cells that H3K36me3, together with MutSα, is involved in the protection against mutations, preferentially in actively transcribed genomic regions (Huang et al., 2018). The authors found that H3K36me3 and MutSα are co-enriched in exons and actively transcribed regions rather than in introns and non-transcribed regions. Depleting the H3K36me3 or disrupting the H3K36me3-MutSα interaction elevated spontaneous mutation frequency in actively transcribed genes, but only had a minor impact on the mutation rate in non-transcribed or transcriptionally inactive regions. This suggests that H3K36me3-mediated MMR protects actively transcribed genes by removing DNA lesions associated with a persistently open chromatin structure. A connecting link in plants between H3K36me3, or any histone mark, and MMR has not yet been identified but cannot be excluded. In summary, it seems that the protection of genic regions during DNA replication by the MMR system against an excessive accumulation of mutations is a conserved mechanism.

## Induced Mutagenesis Leads to an Increased Mutational Frequency

Naturally occurring mutations are unpredictable changes in DNA caused due to errors in replication or through environmental factors such as sunlight. In spontaneous mutations, the source of the mutation is either not predictable or unknown. Thus, determining the specific cause of a spontaneous, natural mutation, either through DNA replication or through environmental factors, is not possible. Induced mutations are caused by exposure to physical or chemical agents, for example, as applied in mutagenesis breeding or in basic research. For many decades, plant breeders have used chemical mutagens or irradiation to increase the frequency of the formation of mutations in seeds or other parts of plants (Tadele, 2016). The concentration of the mutagen, the length of treatment, and the temperature at which the experiment is carried out affect the efficiency of mutagenesis. The concentration of the mutagen that results in the best mutational frequency with the least possible unintended damage is considered the optimal dose for induced mutagenesis (Mba et al., 2010). The most commonly used chemical substance in plant breeding is ethyl methanesulfonate (EMS; Sikora et al., 2011). EMS selectively alkylates guanine bases forming O6-ethylguanine, which base pairs with thymine, causing a base mispairing. It has been shown that the treatment of the cells with EMS primarily induces point mutations in the genome with a bias for G/C to A/T mutations in many plants (Greene et al., 2003; Till et al., 2004, 2007). Beside its function in repairing replication errors, the MMR system also recognizes and repairs sites of alkylation damage (Wang and Edelmann, 2006). Depending on the doses of EMS, the sites of damage can be larger than the number of available components of the MMR and some damage might remain unrepaired. The induced mutations might also be repaired at different rates depending on their local environment (Marsischky and Kolodner, 1999; Greene et al., 2003). Overall, EMS treatment leads to an increased mutational frequency that enhances genetic variation. Mutagenized plants are screened phenotypically as either a forward genetic approach or point mutations can be identified using a reverse genetic strategy that is known as Targeting Induced Local Lesions In Genomes (TILLING; McCallum et al., 2000). After treatment with the mutagenic agents, the seeds are planted and allowed to self-fertilize to produce a T2 generation. DNA from T2 plants is isolated and screened for desired mutations (Sikora et al., 2011). Ordinarily, TILLING uses a mismatch-specific endonuclease to identify induced point mutations in populations of mutagenized individuals (Colbert et al., 2001). Next-generation sequencing is gradually replacing classical TILLING for mutation screening as it is a faster and more efficient approach (Gupta et al., 2017). After the identification of a plant carrying a beneficial mutation, undesired changes of the DNA are outcrossed (Kurowska et al., 2011). This high-throughput screening for induced point mutations is a very efficient and fast approach to identify mutations in a gene of interest. EMS-saturated mutagenesis screens combine EMS treatment with TILLING and are being used to identify all genes that are involved in certain biological processes. EMS-saturated mutagenesis screens were applied, for example, in *Arabidopsis thaliana* to identify mutations that result in herbicide-resistant plants (Jander et al., 2003). Induced mutagenesis by EMS treatment results in a specific mutational pattern, but the parts of the genome where these mutations occur and if they are repairable by cellular repair mechanisms are not predictable. On the other hand, CRISPR/Cas9 enables in a site-directed process precise changes of the genome at targeted sites. Recently, it was shown that in human cells, Cas9-induced DSBs tend to be predominantly repaired in an error-prone way, leading to the introduction of genomic alterations rather than recovering the original DNA sequence (Brinkman et al., 2018). Their results indicate that the error rate of the repair of Cas9-induced DSBs is locus dependent. The estimated error rates of 20–100% per break event induced by Cas9 appear rather high, suggesting that the repair of Cas9-induced DSBs is not representative for the repair of naturally occurring DSBs (Brinkman et al., 2018). The enhanced repair error rates of Cas9-induced DSBs and the targeting of Cas9 to any specific target regions increase the likelihood of mediating an alteration of the DNA sequence enormously, even in naturally rather less accessible parts of the genome.

## Conclusion

The major aim in plant breeding is to develop and improve plants by altering specific traits to increase their yield and quality. The basis for the development of new cultivars in conventional plant breeding is genetic variation. Genetic variation occurs naturally due to spontaneously emerging mutations and meiotic recombination or can be induced by mutagenesis. DNA damage can appear randomly at any part of the genome subsequently leading to mutations. Cellular mechanisms stop the progression of the cell cycle where DNA damage is sensed and also activate repair mechanisms to protect distinct areas of the genome to sustain genomic integrity. In some cases, certain parts of the genome are more protected than others. For example, the DNA mismatch repair (MMR), which is, among others, activated by mistakes during replication, especially protects genic regions in the genome of *Arabidopsis thaliana* against newly occurring mutations (Belfield et al., 2018). The protection of actively transcribed genic regions by the MMR was also shown in bacteria, yeast, and human cells (Lee et al., 2012; Supek and Lehner, 2015; Sun et al., 2016; Foster et al., 2018; Huang et al., 2018). New genome editing technologies, such as CRISPR/Cas9, are now making the entire genome accessible for any desired change by the researcher and breeder. These new techniques circumvent mechanisms that protect certain areas of the genome by targeting nucleases to specific genomic regions, thereby increasing the probability of the induction of genomic alterations. Kinetics and fidelity studies showed that the repair of Cas9-induced DSBs is not representative for the repair of naturally occurring DSBs indicating that natural processes are bypassed (Brinkman et al., 2018).

The attempt to mutagenize any part of the genome started with the development of EMS screens in order to obtain mutants that carry mutations in all genes relevant for a specific biological process of a plant. The intention of this reverse genetics approach is to test the function of specific genes by analyzing the consequences of their disruption on the process of interest. Recently, for example, an EMS screen in *Arabidopsis thaliana* was applied to generate a mutant collection in order to identify genes that lead to meiotic defects (Capilla-Perez et al., 2018). Mutant collections are very useful resources for functional genomics and basic research in *Arabidopsis thaliana*. Nevertheless, the outcome of these screens, i.e., the exact genomic region where a new mutation originates, is not predictable or determinable. By contrast, CRISPR/Cas allows the mutation of any genomic region of interest, given that a PAM sequence is present around the target region depending on the Cas variant that is used. With this tool, researchers and breeders are no longer dependent on unpredictably occurring mutations, and it allows highly flexible editing of the genome.

Meiosis is a naturally occurring process leading to a new combination of parental DNA. This exchange of chromosome segments between non-sister chromatids during the production of gametes takes place at defined regions of the chromosomes. Breeders use meiotic recombination during backcrossing to combine genetic material that is desirable for agriculture. However, some crop genes lie in low-recombinogenic regions of the chromosomes, which mean that some parts of the genome are not easily or not at all accessible for replacement of certain alleles. Examples of plant species that have large blocks of linked genes are barley and tomato. Site-directed nucleases, such as CRISPR/Cas9, can replace alleles in recombinogenic cold spots fast, easily, and precisely leading to a combination of genetic material that would hardly occur naturally, thereby overcoming these natural limitations.

CRISPR/Cas9 is a technology that allows the rapid generation of genetic changes in specified target regions of the genomes of many organisms *via* the NHEJ repair and the integration of DNA sequences in a homology-directed manner by HDR. The restriction of CRISPR/Cas9 due to its PAM requirement at the target sequence is constantly being reduced as many further alternatives to the classical Cas9-system are discovered and developed. The target sequence is recognized by an individually synthesized gRNA together with Cas9, leading to an activated nuclease function of Cas9. Many plants have highly redundant genomes, which mean that they have many genes in multiple copies and most genes can be grouped in gene families based on sequence similarities. Gene family size is variable across different species and may have important functional outcomes related to speciation (Nei and Rooney, 2005; Woodhouse et al., 2011). Furthermore, many plant species are polyploid, meaning that they have more than two paired (homologous) sets of chromosomes. The gRNAs are capable of recognizing all complementary target sequences present in the genome, irrespectively of how many gene copies are present. That means it is possible to alter at the same time all or multiple gene copies carrying the same target sequence in an organism. These alterations are obtainable neither using standard breeding techniques nor through naturally occurring mutations. Cas9 also enables the editing of different genomic sites using different gRNAs in one organism simultaneously or successively in multiplexing approaches. This, together with the aforementioned, allows the site-directed editing of closely situated base pairs, which is not possible through conventional breeding. In summary, the outcomes of these editing processes can result in organisms with new genetic combinations that would not occur naturally. Comparing all genomic alterations or allelic combinations generated by CRISPR/Cas9 generally as identical to naturally occurring variations is a misleading oversimplification.

## Author Contributions

KK planned and wrote the manuscript.

### Conflict of Interest Statement

The author declares that the research was conducted in the absence of any commercial or financial relationships that could be construed as a potential conflict of interest.
